# Autoimmune Diabetes Is Suppressed by Treatment with Recombinant Human Tissue Kallikrein-1

**DOI:** 10.1371/journal.pone.0107213

**Published:** 2014-09-26

**Authors:** Lilia Maneva-Radicheva, Christina Amatya, Camille Parker, Jacob Ellefson, Ilian Radichev, Arvind Raghavan, Matthew L. Charles, Mark S. Williams, Mark S. Robbins, Alexei Y. Savinov

**Affiliations:** 1 Sanford Project/Children’s Health Research Center, Sanford Research, Sioux Falls, South Dakota, United States of America; 2 DiaMedica USA, Inc., Minneapolis, Minnesota, United States of America; Bristol Heart Institute, University of Bristol, United Kingdom

## Abstract

The kallikrein-kinin system (KKS) comprises a cascade of proteolytic enzymes and biogenic peptides that regulate several physiological processes. Over-expression of tissue kallikrein-1 and modulation of the KKS shows beneficial effects on insulin sensitivity and other parameters relevant to type 2 diabetes mellitus. However, much less is known about the role of kallikreins, in particular tissue kallikrein-1, in type 1 diabetes mellitus (T1D). We report that chronic administration of recombinant human tissue kallikrein-1 protein (DM199) to non-obese diabetic mice delayed the onset of T1D, attenuated the degree of insulitis, and improved pancreatic beta cell mass in a dose- and treatment frequency-dependent manner. Suppression of the autoimmune reaction against pancreatic beta cells was evidenced by a reduction in the relative numbers of infiltrating cytotoxic lymphocytes and an increase in the relative numbers of regulatory T cells in the pancreas and pancreatic lymph nodes. These effects may be due in part to a DM199 treatment-dependent increase in active TGF-beta1. Treatment with DM199 also resulted in elevated C-peptide levels, elevated glucagon like peptide-1 levels and a reduction in dipeptidyl peptidase-4 activity. Overall, the data suggest that DM199 may have a beneficial effect on T1D by attenuating the autoimmune reaction and improving beta cell health.

## Introduction

The two major forms of diabetes mellitus, type 1 and type 2 (T1D and T2D respectively), affect more than 380 million people worldwide [1]. Approximately 5–10% of diabetic patients are afflicted with T1D [2]. Recent epidemiological studies indicate that the world-wide incidence of T1D has been increasing by 2–5% annually [3].

T1D is an autoimmune disease for which there are few therapeutic options other than life-long insulin injections [4]. Insulin administration, however, does not prevent T1D patients from eventually developing co-morbidities such as retinopathy, nephropathy and cardiovascular disease [2]. Novel therapies to address the underlying autoimmune cause of T1D are an urgent unmet need.

The serine protease tissue kallikrein-1 (KLK-1) and its cleavage products lys-bradykinin and bradykinin, are critical components of the kallikrein-kinin system (KKS) [5], [6]. The KKS exerts physiological effects through binding of kinin peptides to the bradykinin 1 and bradykinin 2 receptors [7], [8]. In addition to the blood pressure lowering effects to balance the renin-angiotensin system [9], the KKS is proposed to improve insulin sensitivity [10], [11].

KKS activity has been associated with both positive [12] and negative effects [13] in certain autoimmune diseases. Although there is evidence that administration of porcine and rat tissue kallikrein-1 possess beneficial immune-modulating properties [14], no report to date has investigated the effect of administration of human tissue kallikrein-1 protein in an autoimmune T1D model.

The current exploratory study was designed to evaluate the effects of recombinant human KLK-1 (DM199) protein on the autoimmune progression of T1D in the non-obese diabetic (NOD) mouse. The NOD mouse has been used extensively in T1D studies with a specific focus on the role of T cell-mediated autoimmunity [15]. Two populations of T cells are particularly relevant to T1D pathogenesis. CD8^+^ cytotoxic T cells (CTLs) are primarily responsible for the killing of insulin-producing beta cells [16], whereas the CD4^+^CD25^+^Foxp3^+^ T regulatory cells (Tregs) suppress the activity of CTLs and attenuate the autoimmune attack [17]. Therapies designed to decrease CTL activity and/or increase activity of Tregs, may be effective in treating T1D [18], [19]. Here, we show that chronic treatment of NOD mice with DM199 delays the onset of T1D and attenuates the autoimmune response as evidenced by modulation of the relative populations of CTLs and Tregs in the pancreas and pancreatic lymph nodes. The resulting protection of insulin-producing beta cells was associated with DM199 dose-specific improvements in whole-body glucose disposal, serum C-peptide and glucagon like peptide-1 (GLP-1) levels, and inhibition of serum dipeptidyl peptidase-4 (DPP-4) activity.

## Materials and Methods

### Reagents

All chemicals were purchased from Fisher Scientific (Suwanee, GA), unless stated otherwise.

### Recombinant DM199 preparation

DM199 was produced from Chinese hamster ovary (CHO) cells expressing a gene encoding the full-length pre-pro-protein for human tissue kallikrein-1 (NP_002248.1). Following harvest and clarification, the supernatant containing secreted pro-KLK-1 was treated with recombinant trypsin (Roche Diagnostics, Germany) to generate active KLK-1. The active KLK-1 protein (DM199) was purified under aseptic conditions through multiple column chromatography and filtration steps as previously described [20]. Briefly, trypsin-digested KLK-1 was purified through an Octyl Sepharose 4 FF column, followed by affinity purification on a Benzamidine Sepharose FF column. Following buffer exchange, the eluate was purified through a DEAE Sepharose column (all columns were from GE Healthcare, Piscataway, NJ). The DEAE Sepharose eluate was concentrated, buffer exchanged into PBS (pH 7.4) and sterile-filtered. DM199 was >95% pure by densitometric analysis of SDS-polyacrylamide gels, with <0.5 EU/ml endotoxin levels by the LAL endotoxin test.

The specific activity of DM199 was measured *in vitro* by cleavage of the substrate D-Val-Leu-Arg-7 amido-4-trifluoromethylcoumarin (D-VLR-AFC, FW 597.6) (Sigma-Aldrich, St. Louis, MO or AnaSpec Inc., Freemont, CA). When D-VLR-AFC was hydrolyzed, the free AFC produced in the reaction was quantified by fluorometric detection (excitation 360 nm, emission 460 nm). DM199 activity was determined by comparing the relative activity of a DM199 sample to the porcine kininogenase standard acquired from the National Institute for Biological Standards and Control (NIBSC Product No. 78/543). For this standard, the assigned potency is 22.5 international units (IU) per 20 µg ampoule of porcine pancreatic kininogenase.

### Animal Research & Ethics Statement

This study was carried out in strict accordance with the recommendations of the Guide for the Care and Use of Laboratory Animals of the National Institutes of Health. All animal procedures were approved by the Sanford Research Intuitional Animal Care and Use Committee (Protocol #22-12-10-13B). Animals were housed in the Sanford Research Laboratory Animal Facility with food and water provided *ad libitum*. All efforts were made to minimize animal suffering during the experiments. At the end of each experiment, animals were euthanized by CO_2_ asphyxiation.

### Animals and experimental animal procedures

Female NOD/ShiLtJ (NOD) mice were purchased from the Jackson Laboratory (Bar Harbor, ME) at 4 weeks of age. Treatment was initiated in NOD mice at 6 weeks of age. Animals were screened for urinary glucose using Bayer Diastix strips (Tarrytown, NY) twice-weekly for the first six weeks of treatment; thereafter the frequency of testing was increased to daily. In animals with a positive urine glucose test, non-fasting blood glucose was measured using a Bayer Ascensia Elite 1 one-touch blood glucose monitor (Tarrytown, NY). The onset of diabetes was defined as non-fasting blood glucose concentrations greater than 250 mg/dL for two consecutive days.

Effects of DM199 were studied in three consecutive experiments. In the first experiment (Cohort 1), 6 groups were treated as per Table 1 for up to 18 weeks, or until the onset of diabetes. In this experiment a subset of animals from groups 1–4 were sacrificed after 4 weeks of treatment and evaluated for the formation of insulitis. Newly diabetic animals were sacrificed immediately, while all remaining animals were sacrificed at the end of the 18-week treatment period. Upon sacrifice, spleens, pancreatic lymph nodes and pancreata were removed and processed for analyses.

**Table 1:** DM199 dosing regimen and study cohort details.

Dose Group^1^^,^ 2 ^,^ 3	DM199 dose (Units/kg)	Treatment Frequency	Treatment group name
1	None	Daily	Vehicle
2	2	Daily	2 U/kg/day
3	40	Daily	40 U/kg/day
4	100	Daily	100 U/kg/day
5	100	Thrice weekly	100 U/kg/3x-week
6	100	Once weekly	100 U/kg/1x-week

1 Cohort 1: All dose groups; 18-week treatment.

2 Cohort 2: All dose groups; 10-week treatment.

3 Cohort 3: Dose groups 1 & 4; 9-week treatment.

In the second experiment (Cohort 2), 6 groups of animals were treated (Table 1) for up to 10 weeks, or until the onset of diabetes. All non-diabetic animals still remaining in the study after 9 weeks of treatment received i.p. injections of synthetic nucleotide EdU for 5 consecutive days for postmortem assessment of β cell proliferation. At the end of 10 weeks all animals were sacrificed, spleens, pancreatic lymph nodes and pancreata were removed and processed for analyses.

In the third experiment (Cohort 3), 2 groups of animals were injected daily with either DM199 at 100 U/kg or PBS for 9 weeks, for measurement of active GLP-1 levels and DPP-4 activity in sera.

### Safety markers - blood pressure and toxicology

Blood pressure measurements were performed on randomly selected mice from each group in Cohort 1 using a NIBP Multi Channel Blood Pressure System (IITC Life Science Inc. Woodland Hills, CA). Animals were acclimated to the restrainers for several days prior to data collection. Animals were placed into the analyzer restrainer immediately after treatment, tail cuffs with pulse wave detection sensors were placed on restrained animals, and the blood pressure parameters were measured.

All animals were monitored weekly for overall health, behavior, and bodyweights for potential toxicological effects. Post mortems on Cohort 1 were conducted for visual signs of organ pathology and confirmed by histopathology assessment of liver, heart, kidney and brains after 18 weeks of treatment.

### Intraperitoneal glucose tolerance tests (IPGTT)

Subgroups of NOD mice from cohort 1 were fasted overnight, injected with 2.5 g/kg of glucose in PBS intraperitoneally (i.p.), and blood glucose was measured at 0, 20, 40, 60, 90, and 120 minutes after glucose challenge. Blood was collected via tail vein into heparinized capillary tubes and blood glucose measured with a Bayer Ascensia Elite 1 one-touch blood glucose monitor.

### ELISA measurements of GLP-1, C-peptide, insulin, total and active TGF-β1 levels

During the in-life portion of each experiment, sera were collected from 100–200 µl of blood withdrawn from the retro-orbital venous sinus of each non-diabetic animal. At the end of the in-life portion of each experiment, sera were collected from blood withdrawn from the hearts of animals immediately after sacrifice. For evaluation of active GLP-1, blood samples were collected in the presence of a DPP-4 inhibitor (Millipore, St. Charles, MO). All samples were stored at −80°C and then analyzed by ELISA-based kits for the determination of mouse insulin (Millipore), active GLP-1 (ALPCO Salem, NH) C-peptide (ALPCO), total TGF-β1 (R&D Systems, Minneapolis, MN), and active TGF-β1 (BioLegend), according to the manufacturer’s instructions.

### Assessment of DPP-4 activity in sera

Sera samples were collected from Cohort 3 animals after nine weeks of treatment and were stored at −80°C. Serum DPP-4 activity was determined by monitoring the proteolysis kinetics of the synthetic substrate (H-Gly-Pro-AMC, R&D Systems) that generates a fluorogenic peptide. Serum samples (10 µl) were diluted in 25 mM Tris (pH 8.0) and the substrate (20 µM) was added to the reaction mixture to a final volume of 100 µl. Reactions were read at excitation and emission wavelengths of 380 nm and 460 nm, respectively, in kinetic mode for 60 minutes using a SpectraMax M5 microplate reader (Molecular Devices, Sunnyvale, CA).

### Immunohistochemical studies

Pancreata were weighed, embedded into OCT compound (Fisher Scientific), flash-frozen, and cryo-sectioned into 5 µm-thick sections for placement on Superfrost glass slides. Slides were fixed with 100% acetone at −20°C for 5 min, air-dried and stored at −80°C until staining. For insulin immunostaining with Hematoxilin and Eosin (H&E) counter-stain, sectioned samples were first stained with rabbit polyclonal anti-insulin antibody (ProteinTech, Chicago, IL) and insulin was detected using the avidin-biotin-peroxidase immunohistochemistry kit LSAB 2, (DAKO, Carpinteria, CA.) according to the manufacturer’s protocol for the 3-amino-9-ethylcarbazole substrate-chromogen solution. Sections were then counter-stained with H&E and visualized under Nikon 90i microscope. For immunofluorescent staining, tissue sections were incubated with 1% BSA in PBS overnight at 4°C with 100 µl/section of guinea pig anti-mouse insulin (ab7842; Abcam, Cambridge, MA), followed by Alexa Fluor 594-labeled donkey anti-guinea pig Fc secondary antibody (Jackson Immunoresearch, West Grove, PA). Slides were mounted with DAPI-containing Vectashield (Vector Laboratories, Burlingame, CA) and examined under a Nikon 90i fluorescent microscope (Nikon Instruments, Melville, NY). Images were captured with a photometric CoolSNAP HQ2 camera, using the Nis-Elements AR software. β cell mass was calculated by measuring the total insulin-positive stained area of each islet on the section, which was then divided by the total pancreas area of the section, and resultant value multiplied by the total pancreas weight. Three slides per mouse on average were analyzed in a blinded fashion, with the sections being a minimum of 150 microns apart.

### Analysis and grading of insulitis

The severity of insulitis was determined on the pancreatic sections fluorescently stained for insulin, as described above (with a minimum of 9 islets/mouse), and graded in a blinded fashion as: Grade 0–intact islets, no infiltrating cells; Grade 1–peri-insulitis, infiltrating leukocytes are located around islet mass, do not penetrate islet “capsule”; Grade 2–insulitis, leukocytes clearly penetrating into islets, reducing β cell mass by about 25%; Grade 3–heavy insulitis, infiltrating leukocytes reduce β cell mass by about 50%; Grade 4–destructive “end-stage” insulitis, where virtually no β cells are left within the islet infiltrate. The overall insulitis score for each group was determined as the average insulitis grade, weighted by the percentage of islets with the observed grade.

### Assessment of beta cell proliferation

Pancreata from animals in treatment Cohort 2 that received EdU injections, were assessed for β cell proliferation *via* detection of EdU incorporation into the DNA of replicating cells, as previously described [21]. Slides containing pancreatic sections were prepared as described under ‘Immunohistochemical studies’, washed in PBS, incubated for 20 min at room temperature with staining mixture (10 µM; Cy5-labeled azide, 1 mM CuSO_4_, 0.1 M ascorbic acid in 100 mM Tris-HCl pH 8.5). After staining, slides were rinsed in TBS with 0.5% Triton X-100, washed, then blocked with 1% BSA, and processed for insulin staining. Beta cell proliferation was determined by counting the EdU and insulin co-positive cells using Nikon NIS Elements and Image J. The proliferation rates were calculated on an average of three slides per mouse, with sections being a minimum of 150 microns apart. A minimum of fifty islets/mouse were analyzed and all slides were analyzed in a blinded fashion.

### Analyses of islet infiltrate composition

Assessment of islet infiltrate composition was performed by immunofluorescent staining of pancreatic sections for CD4 and CD8 T cell surface markers (using an average 15 sections/mouse). Slides were first stained for CD4 using primary rat anti-mouse CD4 (BioLegend, Clone GK1.5) antibody followed by a secondary donkey anti-rat Cy5-labeled detection antibody (Jackson Immunoresearch). After extensive washing, the slides were incubated for 1 hr with FITC-labeled rat anti-mouse CD8 antibody (BioLegend, Clone 53-6.7), washed and mounted in DAPI-containing Vectashield. As absolute numbers of islet-infiltrating T cells can vary widely between individual islets depending on the size of infiltrate, attempts to normalize this variance involved calculating the CD4^+^/CD8^+^ T cells ratios for each islet.

To count the frequency of T regulatory cells within islet infiltrates, indirect Foxp3 staining was performed with rat anti-mouse anti-Foxp3 primary antibody (eBioscience, San Diego, CA, Clone NRRF-30) followed by DyLight594 labeled donkey anti-rat-Ig (Jackson Immunoresearch). Anti-mouse CD4-FITC (eBioscience, Clone RM4-5) was then applied. All antibodies were diluted at 1∶100 for immunostaining.

### Immune cell isolations and FACS analyses

Pancreatic lymph nodes (PLNs) and spleens were mashed in PBS to release single cells into suspension. Single-cell suspensions of splenocytes were exposed to ACK lysis buffer (Fisher Scientific) to eliminate red blood cells, centrifuged and re-suspended in PBS. Cells in all samples were counted with a hemocytometer and diluted with FACS buffer (2% FBS; 0.1% sodium azide in PBS) to a concentration of 1 million cells per 100 µL. The Fc receptors were blocked for 20 minutes on ice with anti-CD16/CD32 block solution (eBioscience) prior to staining of cells with antibodies.

For Treg analyses, single cell suspensions were stained with anti-Foxp3, anti-CD4, anti-CD127, and anti-CD25 fluoro-labeled antibodies. Specifically, cells were incubated on ice with FITC-conjugated anti-CD4 (1∶200; eBioscience, Clone RM4-5), APC-conjugated anti-CD25 (1∶100; eBioscience, Clone PC61.5) and PerCP/Cy5.5-conjugated anti-CD127 (1∶500; Biolegend, Clone A7R34) antibodies for 30 min. The cells were then pelleted at 1,400 rpm, washed twice with FACS buffer and processed for Foxp3 staining using PE-conjugated anti-Foxp3 (1∶40; eBioscience, Clone NRRF-30) according to the manufacturer’s protocol. Cells were finally re-suspended in 200 µL of FACS buffer and analyzed.

For CTL analyses, single cell suspensions from all samples were stained with directly-labeled anti-CD3-PE (1∶300; eBioscience, Clone 145-2C11), anti-CD8-FITC (1∶200; BioLegend, Clone 53-6.7), and anti-CD44-APC (1∶200; Biolegend Clone IM7) antibodies for 30 minutes on ice. Labeled cells were diluted 1∶200 in FACS buffer, centrifuged at 1400 rpm for 5 minutes at 4°C, washed twice with 200 µL FACS buffer, and re-suspended in 200 µL FACS buffer. Live cells were gated using 7AAD (eBioscience) staining. For analysis of activated CTLs, percentages of CD44^+^CD8^+^ cells within the CD3-positive T cell population were calculated. For analysis of naïve CD8^+^ T cell populations in spleens, the percentages of CD44^−^CD8^+^ cells within the CD3-positive population were calculated.

For analysis of cellular DPP-4/CD26 levels, isolated spleens were repeatedly mashed in PBS to release cells. Single-cell suspensions of splenocytes were exposed to ACK lysis buffer (Fisher Scientific) to eliminate red blood cells, centrifuged and re-suspended in PBS. Splenocytes (1×10^5^ cells in 100 µl) were then incubated for 2 h at 37°C in 100 µl RPMI-serum free medium with or without 0.1 U of DM199. Cells were then washed with FACS buffer and blocked with Fc-receptor blocker for 20 min. DPP-4/CD26 was then stained for 30 minutes using FITC-labeled rat anti-CD26 (1∶100; R&D Systems, Minneapolis, MN). Live cells were gated using 7AAD staining.

### Western Blot Analysis

Splenocytes, 5×10^5^, isolated from mouse spleens, were incubated for 2 h at 37°C in 100 µl RPMI serum-free medium supplemented with or without DM199 (0.1 U). Cells were then washed with PBS and lysed in SDS sample buffer. The cell lysates were subjected to electrophoresis through a 4–12% NuPAGE Bis-Tris polyacrylamide gel (Life Technologies, Grand Island, NY) followed by the transfer onto nitrocellulose membrane (Millipore). The membranes were then blocked in 1% Casein in PBST for 1 h followed by overnight incubation with rat monoclonal anti-DPP-4/CD26 antibody (1∶500; R&D Systems). Membranes were washed and treated with HRP-conjugated anti-rat (1∶5000, Jackson Immunoresearch) secondary antibody followed by chemiluminiscent substrate (Super Signal West Dura Extended Duration Substrate, Thermo Scientific, Rockford, IL) for detection according to manufacturer’s instructions. The membrane was scanned on a UVP Biospectrum 500 imaging system (UVP Upland, CA).

### Analysis of Indoleamine 2,3-dioxygenase (IDO) mRNA levels

Dendritic cells were isolated from splenic cell suspension using CD11c Microbeads (Miltenyi Biotec Inc., San Diego, CA) following the manufacturer’s suggested protocol. Total RNA was isolated directly from cells using Direct-zol RNA MiniPrep kit (Zymo Research, Irvine, CA) as described in the manufacturer’s protocol. Residual DNA was removed after on-column DNase treatment. RNA integrity was confirmed on 2100 Bioanalyzer (Agilent Technologies, Santa Clara, CA). 250–1000 ng of RNA was reverse-transcribed using GoScript Reverse Transcription System (Promega Corp., Madison, WI) as described in the manufacturer’s protocol. Aliquots (1 µl) from the reverse transcribed samples were PCR amplified on Stratagene MX3005P using RT^2^ SYBR Green ROX qPCR Mastermix (QIAGEN, Valencia, CA) and primers for beta-actin (QIAGEN), Indoleamine 2,3-dioxygenase (forward primer: 5′-TGGCGTATGTGTGGAACCG-3′ and reverse primer: 5′-CTCGCAGTAGGGAACAGCAA-3′) or CD11c (forward primer: 5′-CTGGATAGCCTTTCTTCTGCTG-3′ and reverse primer: 5′-GCACACTGTGTCCGAAC TCA-3′) genes.

### Statistical analysis

Group statistics were computed using GraphPad Prism (GraphPad Software, La Jolla, CA). Differences in means between groups were analyzed by one-way ANOVA followed by Tukey’s post-hoc tests for significance. Diabetes incidence was plotted as Kaplan-Meier survival curves and significance determined by a log-rank test. Data are presented as mean +/− SEM.

## Results

### Treatment-dependent effects on type 1 diabetes incidence

**Figure 1:**
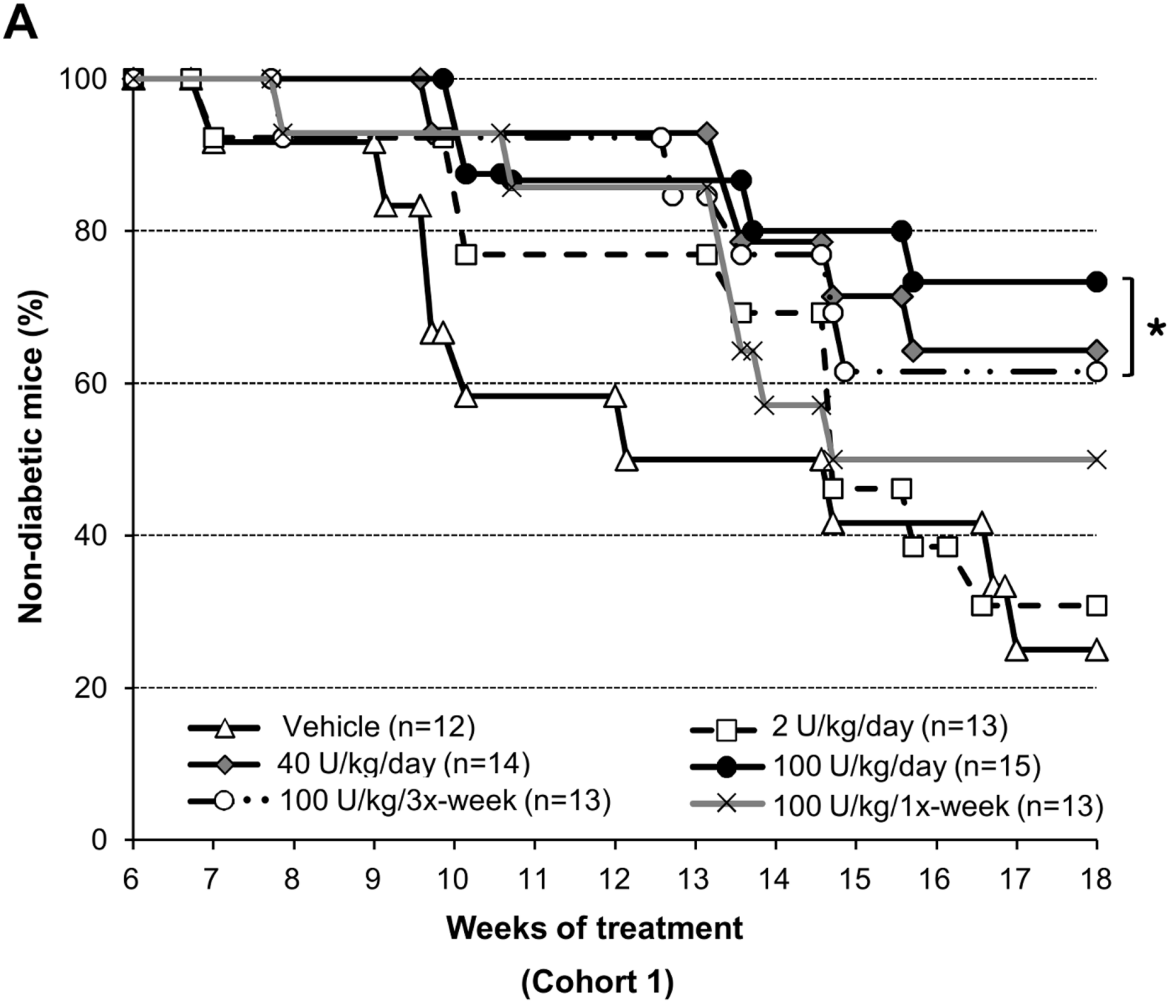
DM199 treatment reduces type 1 diabetes incidence. The fraction of non-diabetic NOD mice remaining over an 18-week treatment period (Cohort 1) is shown. Animals with non-fasting blood glucose >250 mg/dL for two consecutive days were considered diabetic and were removed from the study. None of the mice in any treatment groups developed diabetes in the first 6 weeks of the study. Groups of NOD mice were injected with vehicle or DM199 *i.p*. as indicated in the legend; n, initial number in each group. *P<0.05 in comparison to control using a log-rank test.

The time course of spontaneous T1D development in NOD female mice is reasonably predictable, with the first animals presenting diabetes at approximately 12 weeks of age, and the incidence reaching 60 to 80% by 20 weeks of age [22]–[24]. To investigate the effects of DM199 on the development of T1D, six groups of six-week old NOD female mice were exposed to a dose-ranging and treatment-frequency varying DM199 treatment regimen (as per **Table 1**) for up to 18 weeks. Control animals developed diabetes at the expected rate with incidence reaching 75% by 24 weeks of age. In contrast, DM199 reduced the incidence of disease in a dose and treatment-frequency dependent manner. The 2 U/kg/day and 100 U/kg once-weekly doses had no significant effect on the incidence rate. The 40 and 100 U/kg/day and 100 U/kg tri-weekly doses significantly reduced the incidence of diabetes at the end of 18 weeks of treatment compared to the control group (**Fig. 1**).

DM199 treatment was generally well tolerated. No significant signs of altered general appearance or distress were noted in any mice at any point during any of the studies. Hypoglycemia was not detected in any animal. At 4 and 18 weeks of DM199 treatment, there was no change in either systolic or diastolic blood pressure compared to placebo treated mice (**Fig. S1A**). A small decrease in net weight gain was observed in the animals treated with 100 U/kg/day of DM199 compared to control (**Fig. S1B**). Postmortem examination after 18-weeks of treatment showed no visual signs of internal organ pathology. Histopathological assessment did not reveal any changes in liver, heart, kidney and brain in any of the experimental groups (data not shown).

### DM199 treatment effects on insulitis and β cell mass

**Figure 2:**
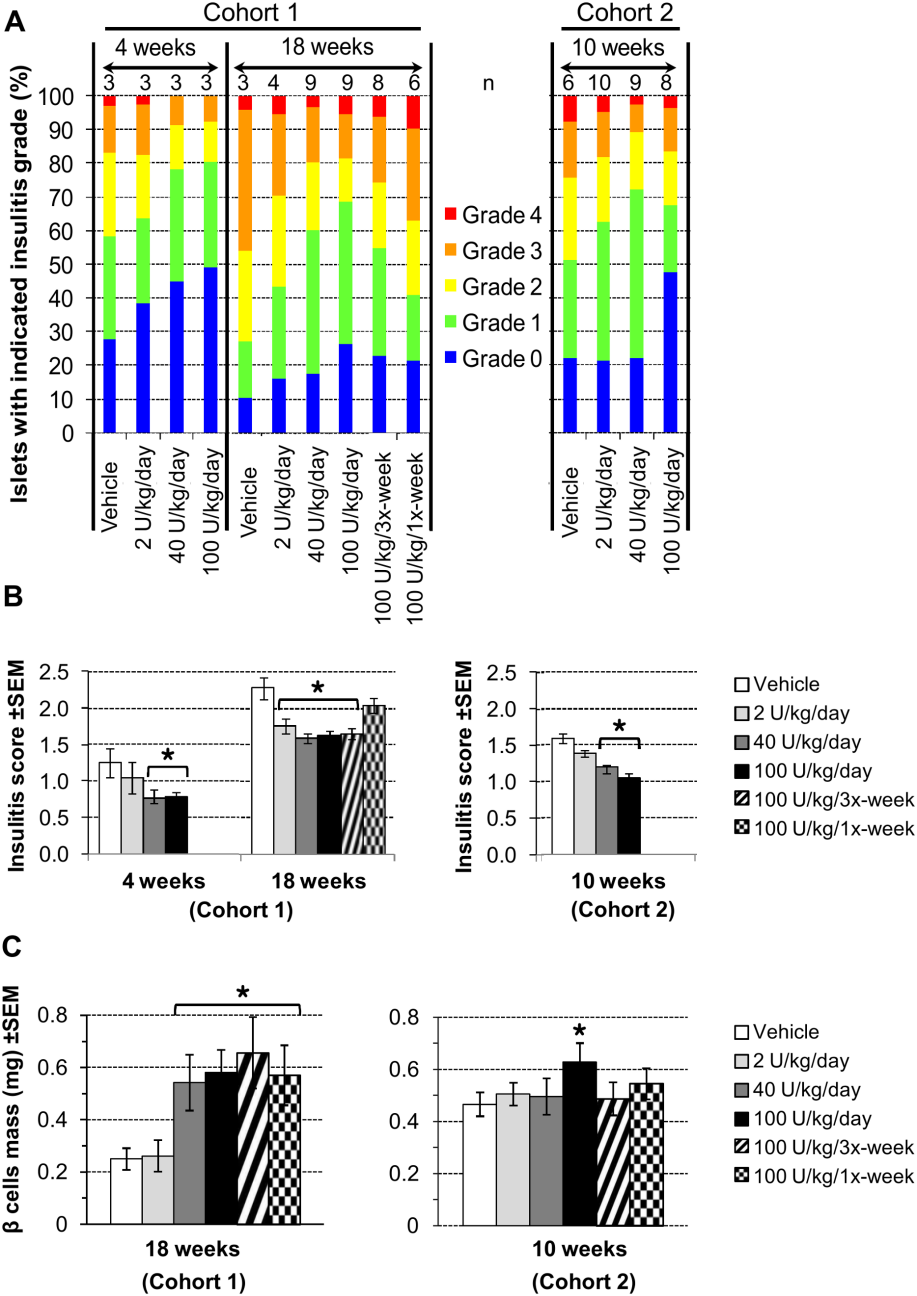
Effects of DM199 administration on insulitis and beta cell mass. Insulitis in pancreatic tissue sections from non-diabetic animals after the indicated treatment periods was graded in a blinded fashion and scored as described in Materials and Methods. Beta cell masses were evaluated as described in Materials and Methods. (A) The proportions of islets in each treatment group with an observed grade; n, number of mice in each treatment group. (B) Average insulitis scores +/− SEM for the two study cohorts. (C) Histomorphometric quantification of pancreatic beta cell mass. *P<0.05 *vs.* control using a one-way ANOVA analysis with Tukey post-hoc multiple comparisons test.

Development of T1D in NOD mice is characterized by autoimmune destruction of the beta cells as evidenced by insulitis and loss of beta cell mass. To elucidate the anti-diabetic effect of DM199, pancreatic islet insulitis was evaluated in subgroups of mice after 4, 10 and 18 weeks of treatment. The degree of infiltration in pancreatic sections was graded, and the average insulitis scores for each group were calculated as previously described [24]. Compared to control, DM199 reduced the proportions of islets with destructive insulitis (grades 2, 3 and 4) for each treatment regimen studied at the indicated time points (**Fig. 2A**). At all time points, the highest percentage of islets with low insulitis grade (0 and 1) was observed with the 100 U/kg/day dose of DM199. After 4 and 10 weeks, mice dosed with 40 and 100 U/kg/day of DM199 showed a significantly lower average insulitis score compared to control mice (**Fig. 2B**). After 18 weeks, all DM199 treatment arms except 100 U/kg/once-weekly showed significantly lower average insulitis scores compared to the control (**Fig. 2B**).

The effects of DM199 treatment on β cell mass and function were assessed on all non-diabetic animals at the end of 18 weeks (Cohort 1) and 10 weeks (Cohort 2) of treatment. Compared to controls, a significantly greater β cell mass was detected in mice receiving 100 U/kg/daily DM199 dose after 10 weeks of treatment, and in mice from all treatment regimens with the exception of the 2 U/kg/day DM199 dose group after 18 weeks of treatment (**Fig. 2C**).

### Physiological response and disease markers

**Figure 3:**
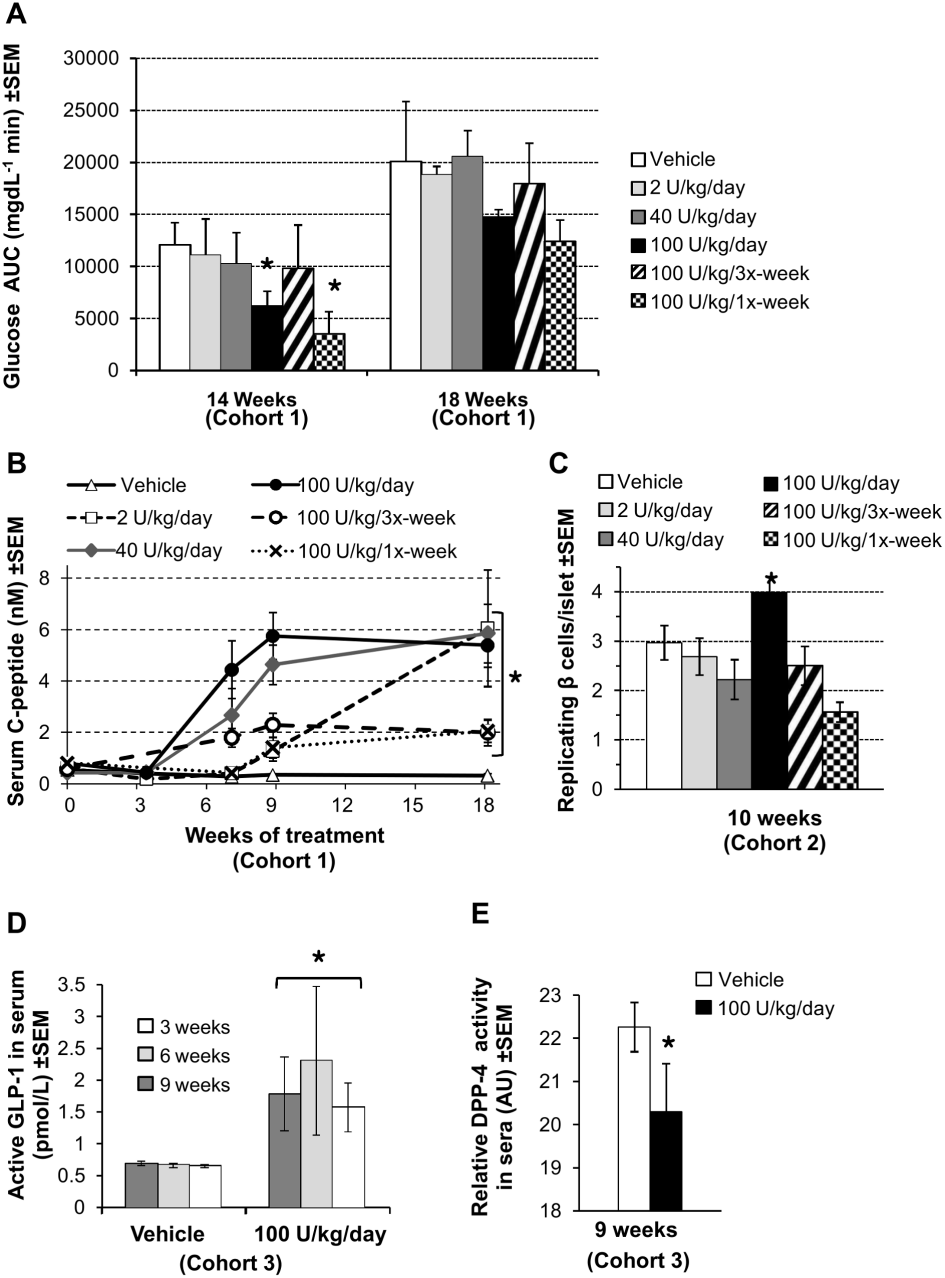
Treatment-dependent anti-diabetic responses. (A) Glucose area under the curves (AUC) during IPGTT (n = 4–7 mice/group) at the indicated time points were calculated from blood glucose readings corrected for fasting blood glucose, and are plotted as the mean +/− SEM. (B) Dose-dependent increase in C-peptide levels. Fasting serum C-peptide concentrations were measured by ELISA (n = 3–11 mice/group) in triplicate at each time point and are represented as mean +/− SEM. (C) Replicating β cell counts in pancreatic islets at 10 weeks. Cells double-positive for insulin and EdU were manually counted as described in Materials and Methods. Data are represented as mean number of replicating β cells per islet ± SEM. (D) DM199 treatment enhances serum GLP-1 levels. Blood samples were collected in triplicate (n = 6–7 mice/group) in the presence of DPP-4 inhibitor and active GLP-1 levels in sera were determined by ELISA. Data represent mean ± SEM. (E) High-dose treatment-dependent reduction in DPP-4 activity. Serum DPP-4 activity was determined by monitoring kinetics of formation of a fluorogenic peptide by proteolysis of H-Gly-Pro-AMC synthetic substrate. Data are represented in arbitrary units (AU) mean ± SEM; (n = 6–7 mice/group). *P<0.05 *vs.* control using a one-way ANOVA analysis with Tukey post-hoc multiple comparisons test.

Having observed a preservation of beta cell mass, we studied other markers associated with glucose control. The physiological response to a glucose load was assessed *via* an intraperitoneal glucose tolerance test (IPGTT) at 14 and 18 weeks of treatment (**Fig. 3A**). At 14 weeks the 100 U/kg/day and 100 U/kg/once-weekly doses significantly reduced the glucose area under the curve (AUC) compared to control. No dose showed any significant improvement at 18 weeks of age and the glucose AUC for all groups appeared to increase by 18 weeks.

Fasting C-peptide and insulin serum levels were measured as indicators of β cell function. [25]. C-peptide levels remained unchanged in all treatment groups during the first 5–6 weeks of treatment. A dramatic increase in fasting C-peptide was observed between weeks 6 and 9 in the 40 and 100 U/kg/day DM199 dose groups. In contrast, only a gradual increase in fasting C-peptide was observed between weeks 6 and 18 for the 2 U/kg/day and 100 U/kg/tri-weekly dose groups. At the end of week 18, C-peptide levels in animals treated with 40 and 100 U/kg/day of DM199 were approximately 12-fold higher compared to control animals (**Fig. 3B**). Fasting serum insulin levels, on the other hand, gradually declined in controls and all treatment groups over the 18-week study period with no difference observed across treatment groups compared to control (data not shown).

To assess if the observed improvement of beta cell mass (**Fig. 2C**) can be at least partially attributed to the DM199-driven β cell proliferation, we examined incorporation of synthetic EdU nucleotide into the DNA of insulin-positive islet cells in Cohort 2 experimental animals after 10 weeks of DM199 treatment (**Fig. 3C**). The averaged numbers of insulin and EdU double-positive cells were between 1.5 and 3 per islet in animals from all experimental groups except mice treated with DM199 at 100 U/kg daily which displayed mild but significant increase in β cell proliferation.

The incretin hormone GLP-1 and its inactivating enzyme DPP-4 are two well-documented targets for intervention in both T1D and T2D. Elevated GLP-1 levels have been reported to be associated with increased β cell mass in a variety of experimental systems [26], [27], and GLP-1 is rapidly inactivated by DPP-4 [28]. We therefore measured serum levels of active GLP-1 and serum DPP-4 activity in mice treated with 100 U/kg/day of DM199. At 3, 6, and 9 weeks, active GLP-1 levels were significantly higher in the treated mice compared to controls (**Fig. 3D**), while serum DPP-4 activity was significantly reduced after 9 weeks in treated mice compared to controls (**Fig. 3E**). We hypothesized that elevated GLP-1 levels could be due in part to DM199-medated proteolytic destabilization of DPP-4. We analyzed the *in vitro* effect of DM199 treatment of splenocytes expressing DPP-4 and showed that DM199 substantially reduced both cell-surface and total cell-associated levels of membrane-tethered isoform of DPP-4, also known as CD26 (**Fig. S2**).

### Modulation of CD8^+^ and Treg lymphocyte populations

The characteristic features of untreated diabetes progression in NOD mice are the increased infiltration of CD8^+^ cytotoxic T cells into the islets and pancreatic lymph nodes (PLNs) together with a concomitant decrease in the frequency and activity of CD4^+^CD25^+^Foxp3^+^ Tregs in the same compartments [17]. We investigated the effect of DM199 treatment for 10 and 18 weeks on lymphocyte populations in the islets, PLNs and spleens.

**Figure 4:**
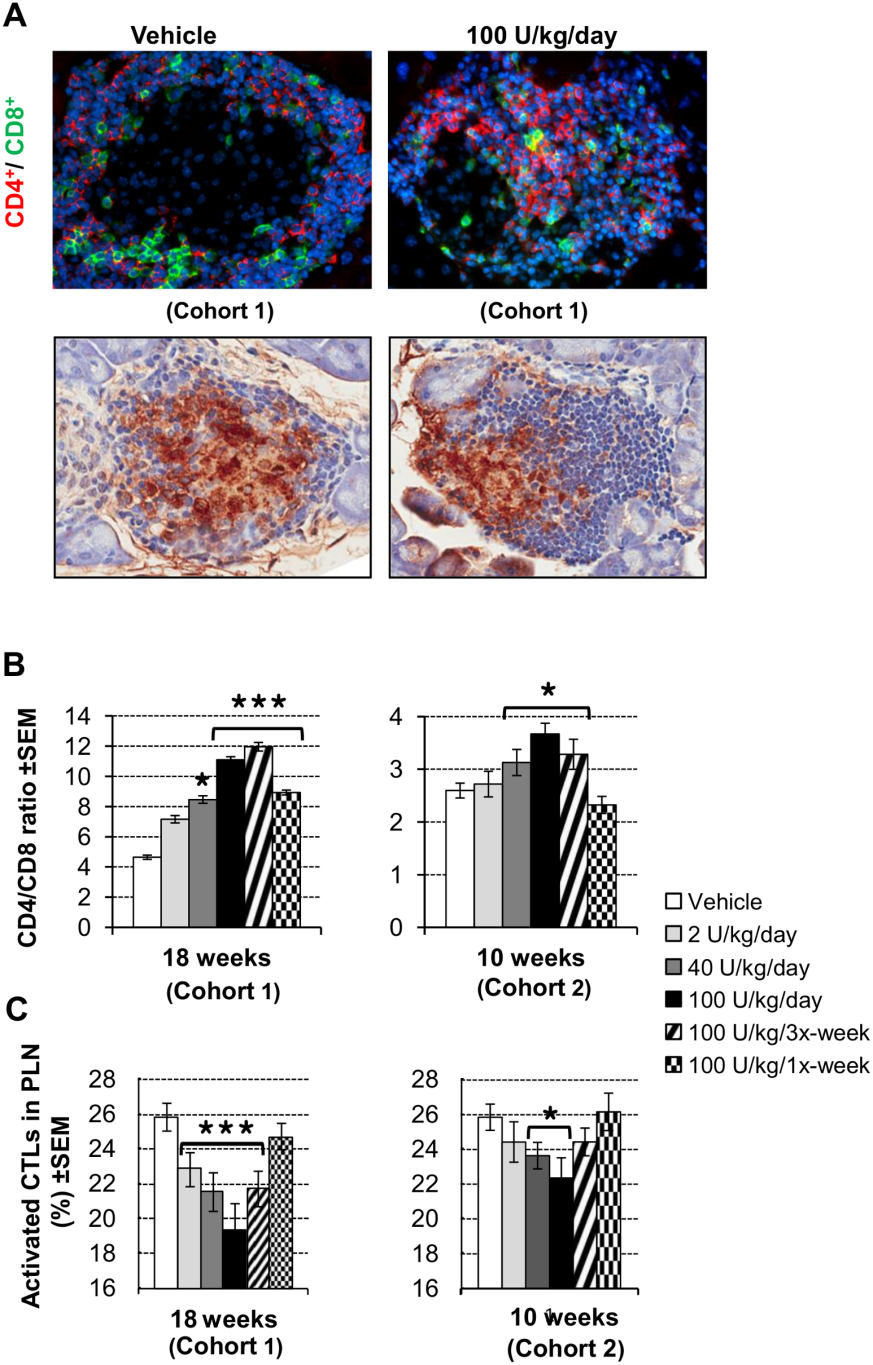
Modulation of CD8^+^ T cell populations. (A) Upper panels: representative images of infiltrated islets stained for CD4 (red), CD8 (green) and DNA (blue) on pancreatic sections from mice treated with either vehicle or 100 U/kg/day of DM199 for 18 weeks. Lower panels: representative images of islets immuno-stained for insulin (brown) and counterstained with H&E. (B) CD4^+^/CD8^+^ ratios in islets. Pancreatic sections from all treatment groups at the indicated time points were fluorescently stained for CD4 and CD8; positive cells were manually counted in a blinded fashion (15 islets/mouse; n = 3–10 mice/group). Data are presented as the mean +/− SEM of the CD4^+^/CD8^+^ ratios for each treatment group. (C) DM199 treatment reduces activated CD8^+^ T cells (CTLs) in pancreatic lymph nodes (PLN). Single cell suspensions of PLN from treated mice were stained against CD3, CD8 and CD44, analyzed by FACS and the percentages of CD44^+^CD8^+^ cells within the CD3^+^ T cell population were calculated. Data are mean ± SEM (n = 4–10 mice/group). *P<0.05 *vs.* control, ***P<0.001 *vs.* control, using a one-way ANOVA with Tukey post-hoc multiple comparisons test.

Immunohistochemical staining followed by morphometric analysis of pancreatic sections was used to evaluate treatment-dependent changes in CD4^+^ and CD8^+^ T cell infiltrate populations. Figure **4A** shows representative images of stained pancreatic islets, indicating a treatment-dependent reduction in the relative number of CD8^+^ T cells and a concomitant increase in the relative number of CD4^+^ T cells. At 18 weeks, treatment with all doses of DM199 except for the 2 U/kg/day dose, significantly increased the CD4^+^/CD8^+^ ratios compared to control (**Fig. 4B**). In Cohort 2 after 10 weeks, significant increases in the CD4^+^/CD8^+^ ratios were observed with the 40, 100 U/kg/day and 100 U/kg/tri-weekly DM199 doses compared to control (**Fig. 4B**). Flow cytometric analysis revealed that the proportion of activated CD44^+^CD8^+^ CTLs in PLNs was significantly decreased at 10 and 18 weeks with 40 and 100 U/kg/day of DM199 treatment compared to control. Significant decreases in the proportion of activated CTLs were also observed at 18 weeks with 2 U/kg/day and 100 U/kg/tri-weekly treatments (**Fig. 4C**). In parallel we observed significant increases in splenic populations of naïve CD44^−^CD8^+^ T cells (**Fig. S3A**) and of CD4^+^CD25^+^Foxp3^+^ Tregs (**Fig. S3B**) after 10 and 18 weeks of DM199 treatment. We also observed significantly elevated expression of indoleamine 2,3 dioxygenase (IDO) mRNA in splenic dendritic cells (**Fig. S3C**).

**Figure 5:**
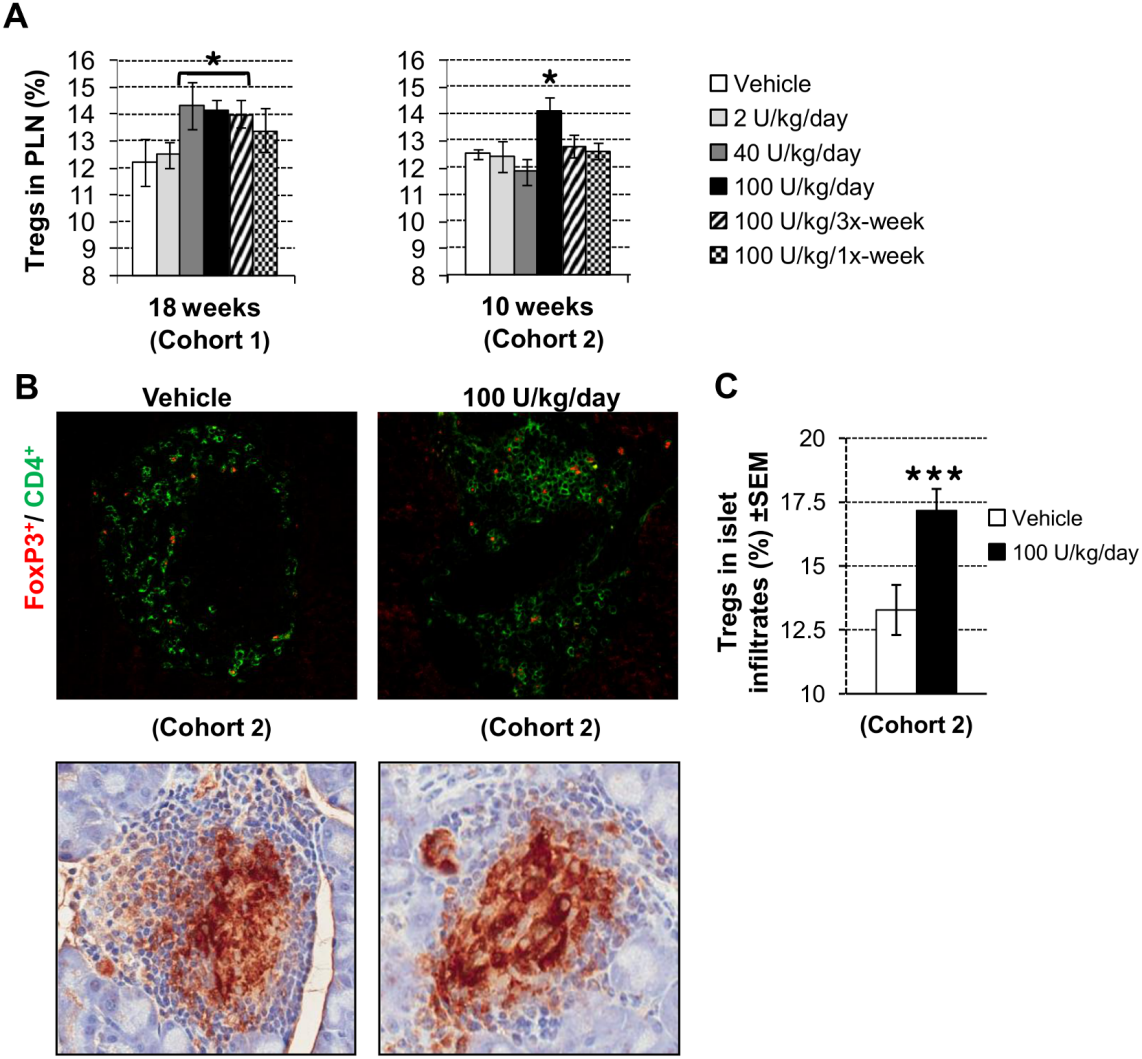
Modulation of regulatory T cell (Tregs) populations. (A) DM199 increases Tregs in pancreatic lymph nodes (PLN). Single cell suspensions of PLN were stained for CD4, CD25, CD127 and Foxp3, and analyzed by FACS. The percentage of CD25^+^Foxp3^+^CD127^−^ Tregs within the total CD4^+^ T cells population was calculated. Data are mean ± SEM (n = 4–10 mice/group). (B) Upper panels: representative images of pancreatic islets stained with anti-Foxp3 (red) and anti-CD4 (green) antibodies, from mice treated for 10 weeks with either vehicle or 100 U/kg/day of DM199. Lower panels: representative images of islets immuno-stained for insulin (brown) and counterstained with H&E. (C) The proportion of CD4^+^Foxp3^+^ Tregs in pancreatic islet infiltrates was calculated for each of at least 40 islets per animal. Data are presented as mean ± SEM (n = 8–9 mice/group). *P<0.05 *vs.* control, ***P<0.001 *vs.* control, using one-way ANOVA with Tukey post-hoc multiple comparisons test.

To assess whether DM199 administration affected the distribution and frequency of Tregs, we evaluated relative numbers of CD4^+^CD25^+^Foxp3^+^CD127^−^ Tregs in the PLNs. At 18 weeks, Tregs levels were significantly higher with the 40 and 100 U/kg/day and 100 U/kg/tri-weekly DM199 dose groups compared to the control (**Fig. 5A**). In Cohort 2, after 10 weeks, only treatment with 100 U/kg/day of DM199 showed a statistically significant increase in the relative PLN Treg population. Representative staining of an islet infiltrate showing CD4^+^Foxp3^+^ T cells after 10 weeks of DM199 treatment is shown in Figure 5B. Analysis of the proportion of Tregs in the islet infiltrates revealed that treatment with the 100 U/kg/day dose of DM199 produced a significant increase in the proportion of Tregs amid the total islet-infiltrating CD4^+^ population (**Fig. 5C**).

### Effect on TGF-β1 levels

**Figure 6:**
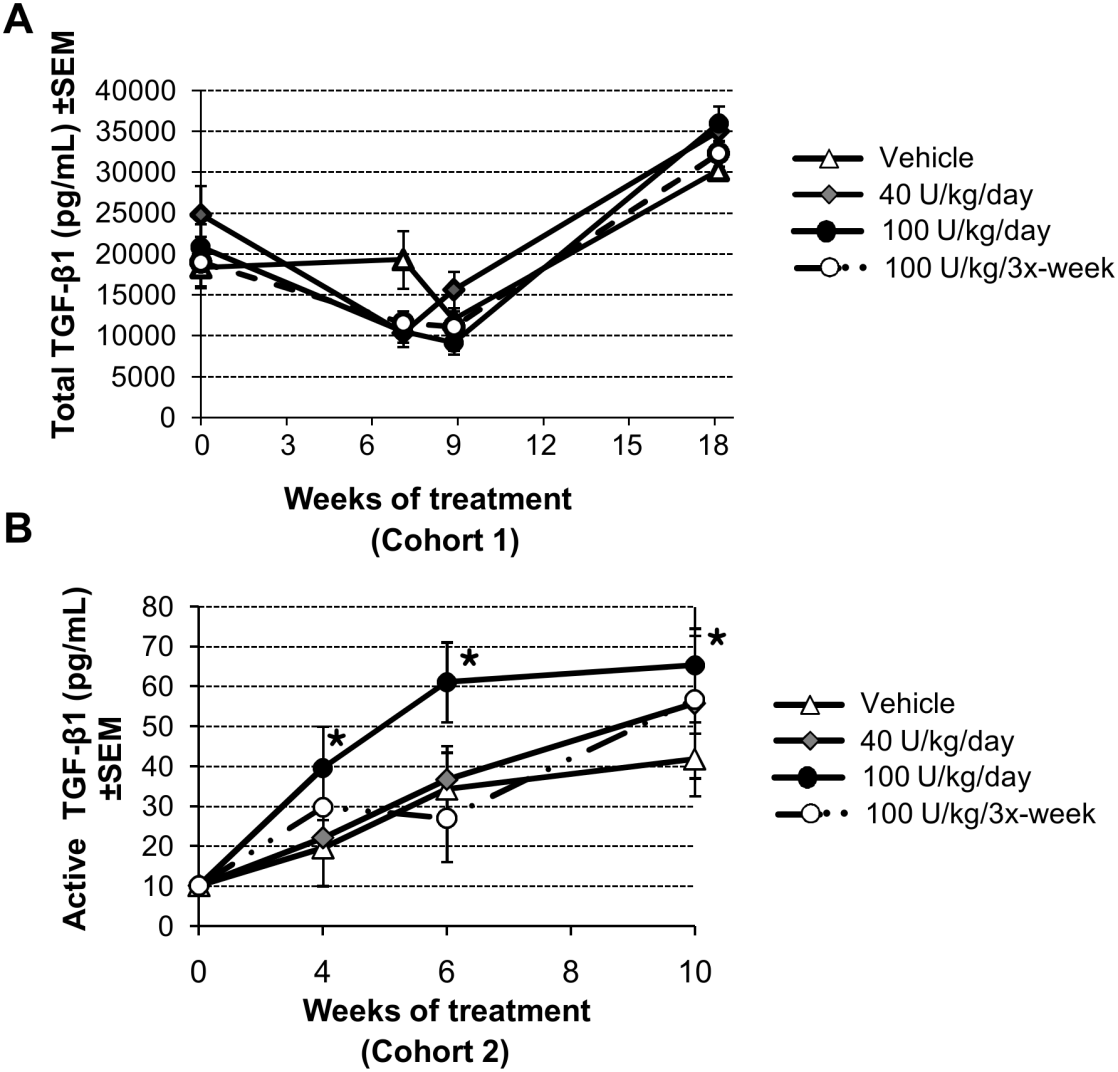
Effect of DM199 on TGF-β1 levels. TGF-β1 levels in sera of mice treated with the indicated DM199 doses were measured by ELISA. (A) Total TGF-β1 from Cohort 1 mice treated for up to 18 weeks. (B) Active TGF-β1 from Cohort 2 mice treated for up to 10 weeks. Data are mean ± SEM (n = 5–7 mice/group). *P<0.05 *vs.* controls, using one-way ANOVA with Tukey post-hoc multiple comparisons test.

Transgenic NOD mice with tissue-specific over-expression of TGF-β1 show a phenotype of increased Tregs and decreased activated CTLs within pancreatic infiltrates and in PLNs [29], [30] Treatment with DM199 resulted in a similar modulation of T cell subpopulations. Therefore, we examined levels of total TGF-β1 in circulation of Cohort 1. No significant differences in total TGF-β1 levels in peripheral blood were detected in any group of experimental animals throughout DM199 treatment (**Fig. 6A**). In a subsequent study (Cohort 2) we examined the effect of DM199 on total and active TGF-β1 levels in sera. Animals treated with 100 U/kg/day of DM199 displayed significant elevation in active TGF-β1 at 4, 6 and 10 weeks compared to controls (**Fig. 6B**).

## Discussion

Human tissue kallikrein-1 (KLK-1) is a ubiquitous serine protease that cleaves kininogen to generate the kinin peptide Lys-bradykinin, which is further processed to bradykinin. Kinin peptides exert effects on several physiological systems including blood pressure regulation, glucose homeostasis [31], cardiac function [32], renal function [33], as well as pain and inflammation [34]. Several reports however, indicate that KLK-1 can mediate these physiologic effects directly and independent of its kininogenase activity [35]–[37].

In autoimmune diseases and T1D, KLK-1 activity has been associated with both beneficial and detrimental effects. Nagy *et al.* reported that KLK-1 suppressed delayed type hypersensitivity in a dermatitis model [14], while Cassim *et al.* suggested that KLK-1 aggravated inflammatory rheumatoid arthritis [13]. In streptozotocin (STZ)-induced diabetic rats, adenoviral KLK-1 gene therapy improved blood glucose levels [38]. The current study examined the preventive effects of chronic administration of recombinant human tissue kallikrein-1 (DM199) in the development of T1D in the NOD mouse model. NOD mice spontaneously develop T1D through autoimmune destruction of β cells, analogous to the mechanism of pathogenesis in humans.

Overall, chronic administration of various doses of DM199 over an 18-week period was well tolerated in animals with no evidence of toxicity, histopathological changes or abnormal physiological responses such as hypotension or hypoglycemia. Tissue kallikrein is best characterized by its vasodilatory effects and delivery of the kallikrein gene in rat models of hypertension elicits long-term reductions in blood pressure [39]. However, in normotensive, myocardial/reperfusion injury rat models, kallikrein gene delivery shows no effect on blood pressure, yet significantly ameliorates other markers of disease progression [40], [41]. We speculate that the lack of a blood pressure-lowering effect (**Fig. S1A**). may be a model-dependent phenomenon and/or a consequence of the method of KLK-1 delivery. The NOD mice in our study are normotensive at 4 weeks and only mildly hypertensive at 18 weeks of DM199 treatment. To the best of our knowledge, only two studies have investigated blood pressure changes following administration of KLK-1 protein. Uehara *et al*. infused sub-depressor doses of rat urinary KLK-1 for 4 weeks in hypertensive rats [42], and Bledsoe *et al*. infused high doses of KLK-1 for 2 weeks in rats with renal injury [43]. In both studies, KLK-1 ameliorated several markers of disease progression, yet no significant effect on blood pressure was observed.

A DM199 dose and treatment frequency-dependent delay in the onset of T1D associated with attenuation of the aggressiveness of insulitis was observed. The attenuation of insulitis corresponded with preservation of beta cell mass with no corresponding increase in insulin and no hypoglycemia. There was an approximate doubling in the IPGTT glucose AUC across all groups between weeks 14 and 18 of the study, which likely reflects a progression of T1D.

The rise in C-peptide levels over the 18-week period observed in all DM199 treatment groups is not clearly understood. C-peptide is a cleavage byproduct of pro-insulin and is secreted in a 1∶1 ratio with mature insulin. C-peptide has a longer serum half-life than insulin and is therefore used as a surrogate marker of insulin levels. We observed a DM199 dose-dependent increase in fasting C-peptide throughout the 18-week treatment period without a concomitant increase in fasting insulin. Although C-peptide and insulin are secreted in a 1∶1 ratio, the disparity between the fasting C-peptide and insulin levels is not surprising. It has been well documented that female NOD mice develop insulin auto-antibodies starting at approximately 5 weeks of age [44]. We hypothesize these auto-antibodies mask circulating insulin from full quantitative detection in our assay. The elevated C-peptide levels correlate with higher beta cell mass and proliferation, and correspond with the delay in the onset of T1D.

GLP-1 and DPP-4 are two well-documented targets for intervention in both T1D and T2D and are potential substrates for DM199 serine proteolytic activity. At the 9-week time point, control animals are beginning to show evidence of hyperglycemia. We noted a significant increase in active GLP-1 levels at 3, 6 and 9-weeks. Therefore we evaluated DPP-4 activity in the high daily dose group at the 9-week time point. We noted a significant decrease in the serum activity levels of DPP-4 that correlated with the observed GLP-1 activity. Further studies on the significance of these effects on DPP-4 and GLP-1 are warranted.

Progression of T1D is characterized by increased pancreatic islet and lymph node infiltration of CTLs and the loss of Treg cell activity [15], [45]. CTLs are effector cells responsible for beta cell destruction leading to loss of glucose control and are an attractive target for treating T1D [18]. Inhibition of CTL migration has been reported to be effective in preventing and treating T1D in the NOD mouse model [46]. Thus we examined the effects of DM199 on CTLs in PLNs and islets.

During T1D pathogenesis, PLNs, and to a certain extent, the spleen, are the major sites of antigenic priming of diabetogenic CTLs. Lower numbers of activated CTLs found in PLNs of animals treated with medium and high daily doses of DM199 may correspond to the less aggressive diabetogenic process and may constitute part of the protective mechanism of DM199 treatment. This suggestion is supported by the demonstration that DM199 treatment reduced the aggressiveness of insulitis. The decrease in the CTLs in PLNs and islets, combined with the increase in naïve CD8^+^ T cells and Tregs in the spleens, suggest that DM199 may be affecting CTL priming, survival, and/or trafficking to the compartments important for the pathogenesis of T1D. More comprehensive studies addressing the direct and indirect effects of DM199 on CTL function are warranted, to better understand the basis for CTL redistribution.

Tregs represent another target for therapeutic intervention [19]. Normalization of the PLN-specific Treg population in diabetic NOD mice was shown to correlate with the recovery of euglycemia, and suggested a potential therapeutic approach for T1D [45]. We observed a marked increase in the relative percentage of Tregs amid the total islet-infiltrating CD4^+^ population and augmented Treg populations in the PLNs of DM199 treated mice. Interestingly, DM199-treated mice showed increased frequencies of Tregs in the spleen and elevated expression of indoleamine 2,3-deoxygenase (IDO) mRNA in splenic dendritic cells. IDO activity has been reported to be associated with maintenance of Treg suppressor state [47], and has also been shown to prevent effective CTL priming [48]. However, the exact relationship between DM199 treatment, splenic Treg populations and IDO activity is at present unclear. Our results suggest that DM199 may be protective by redistributing Tregs between the primary and secondary lymphoid organs.

TGF-β1 is known to play an important role in T1D pathogenesis in NOD mice. Elevated TGF-β1 levels stimulate the expansion of Tregs and enhance their activity [29], [30]. Biologically active TGF-β1 is generated by proteolytic cleavage of a latency-associated peptide from inactive, latent TGF-β1. A recent report suggests that the proteolytic activity of KLK-1 might contribute to activation of latent TGF-β1 *in vitro* [49]. We observed an increase in active TGF-β1 with the highest dose of DM199 (100 U/kg/day) over a 10-week treatment period, and concomitant increases in Treg populations in the spleen and PLNs. We postulate that enhanced levels of the active TGF-β1 cytokine and of CD4^+^ CD25^+^ Foxp3^+^ T suppressor cells, could account for the protective effects of DM199 treatment on the progression of insulitis. Future studies should focus on measuring active TGF-β1 levels over a longer treatment period, more precise characterization of Treg sub-populations, and the proteolytic effects of DM199 on cell surface markers on T cell subpopulations within the spleen, islets and PLNs.

In summary chronic treatment with DM199 in NOD mice was well tolerated, and delayed the onset and reduced the incidence of type 1 diabetes. DM199 appears to have a prophylactic effect on reducing the CTL cell number in the PLN and islets, while also increasing the relative numbers of Tregs in the PLN and splenic compartments. While a detailed analysis of the changes in immune cell function is beyond the scope of this paper, more extensive *in vitro* and *in vivo* studies are needed to delineate the direct and/or indirect immunomodulatory effects of DM199. Analysis of key cytokines such as IL-2, IFNγ, IL-10, IL-17 and TGF-β1, and correlation of their levels to localization of various immune cell sub-populations, could provide a clearer picture of the mechanistic basis of the immunomodulatory effects of DM199. Future studies to address the translatability of DM199 as a potential T1D therapeutic could also include administration of DM199 at the onset or later stages of the disease.

## Supporting Information

Figure S1 **Blood pressure and weight changes following chronic DM199 administration.** (A) The average systolic (*top panel*) and diastolic (*bottom panel*) blood pressure was measured in NOD mice treated with DM199 for 4 or 18 weeks. Results are presented as mean ± SEM (n = 4 mice/group). (B) Average net weight gain in surviving animals treated with DM199 continuously for either 18 weeks (Cohort 1; left panel) or 10 weeks (Cohort 2; right panel). Data are mean ± SEM (n = 3–10 mice/group). *P<0.05 *vs.* control using a one-way ANOVA with Tukey post-hoc multiple comparisons test.
(TIF)

Figure S2 **DM199 reduces cellular CD26 levels.** Isolated splenocytes from mice were incubated for 2 h at 37°C in the absence (Buffer) or presence of 0.1 Units of DM199. (A) Western blot analysis. Splenocyte lysates were subject to electrophoresis, transferred to nitrocellulose membranes and probed with anti-DPP-4/CD26 rat monoclonal antibody. (B) FACS analysis. 1×10^5^ splenocytes treated with buffer or DM199 were stained with either isotype control antibody or FITC-labeled rat anti-CD26 and 7AAD prior to FACS analysis. MFI, mean fluorescence intensity.
(TIF)

Figure S3 **Immunomodulatory effects of DM199 treatment in the spleen.** Single cell suspensions of splenic cells were incubated with ACK lysis buffer to remove erythrocytes, resuspended, stained with antibodies, and analyzed by FACS. (A) The percentage of CD44^−^CD8^+^ cells (naïve CD8^+^ cells) within the CD3^+^ T cell population is represented as mean ± SEM (n = 4–10 mice/group). (B) The percentage of CD4^+^CD25^+^Foxp3^+^ cells (T regulatory cells) within the CD3^+^ T cell population is represented as mean ± SEM (n = 4–10 mice/group). (C) Indoleamine 2,3-deoxygenase (IDO) mRNA expression in splenic dendritic cells after 10 weeks of treatment was determined by qPCR. Data in arbitrary units (AU) normalized to β-actin are represented as mean ± SEM (n = 5 mice/group). **P<0.05; ***P<0.001 *vs.* control using a one-way ANOVA with Tukey post-hoc multiple comparisons test.
(TIF)
