# Supplementary figures and images for: Autoimmune Diabetes Is Suppressed by Treatment with Recombinant Human Tissue Kallikrein-1
Source: PLoS One. 2014 Sep 26;9(9):e107213. doi: 10.1371/journal.pone.0107213 (PMC4178025; doi:10.1371/journal.pone.0107213)

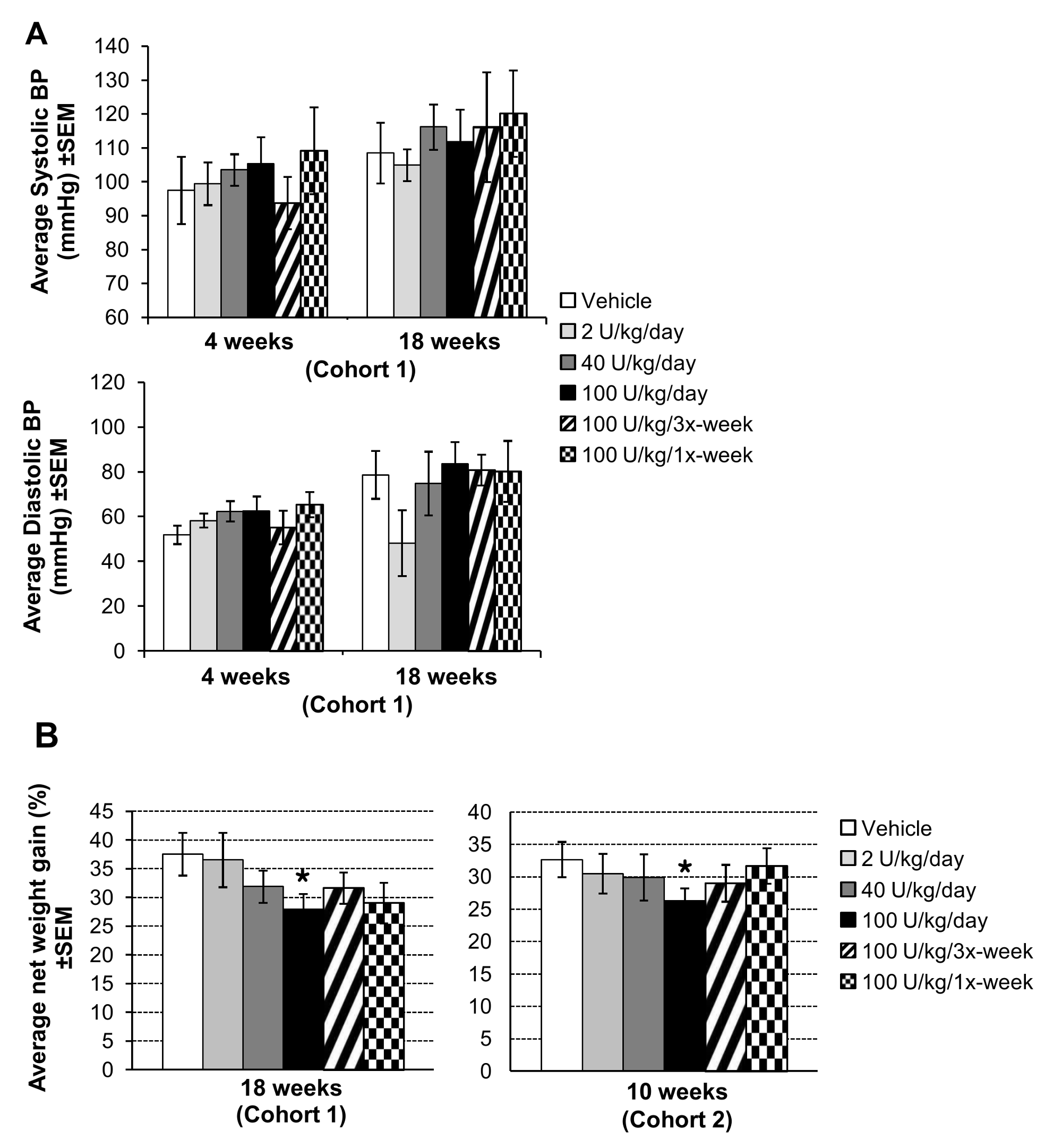

Supplement: Figure S1 — Blood pressure and weight changes following chronic DM199 administration. (A) The average systolic (top panel) and diastolic (bottom panel) blood pressure was measured in NOD mice treated with DM199 for 4 or 18 weeks. Results are presented as mean ± SEM (n = 4 mice/group). (B) Average net weight gain in surviving animals treated with DM199 continuously for either 18 weeks (Cohort 1; left panel) or 10 weeks (Cohort 2; right panel). Data are mean ± SEM (n = 3–10 mice/group). *P<0.05 vs. control using a one-way ANOVA with Tukey post-hoc multiple comparisons test. (TIF) [file pone.0107213.s001.tif]

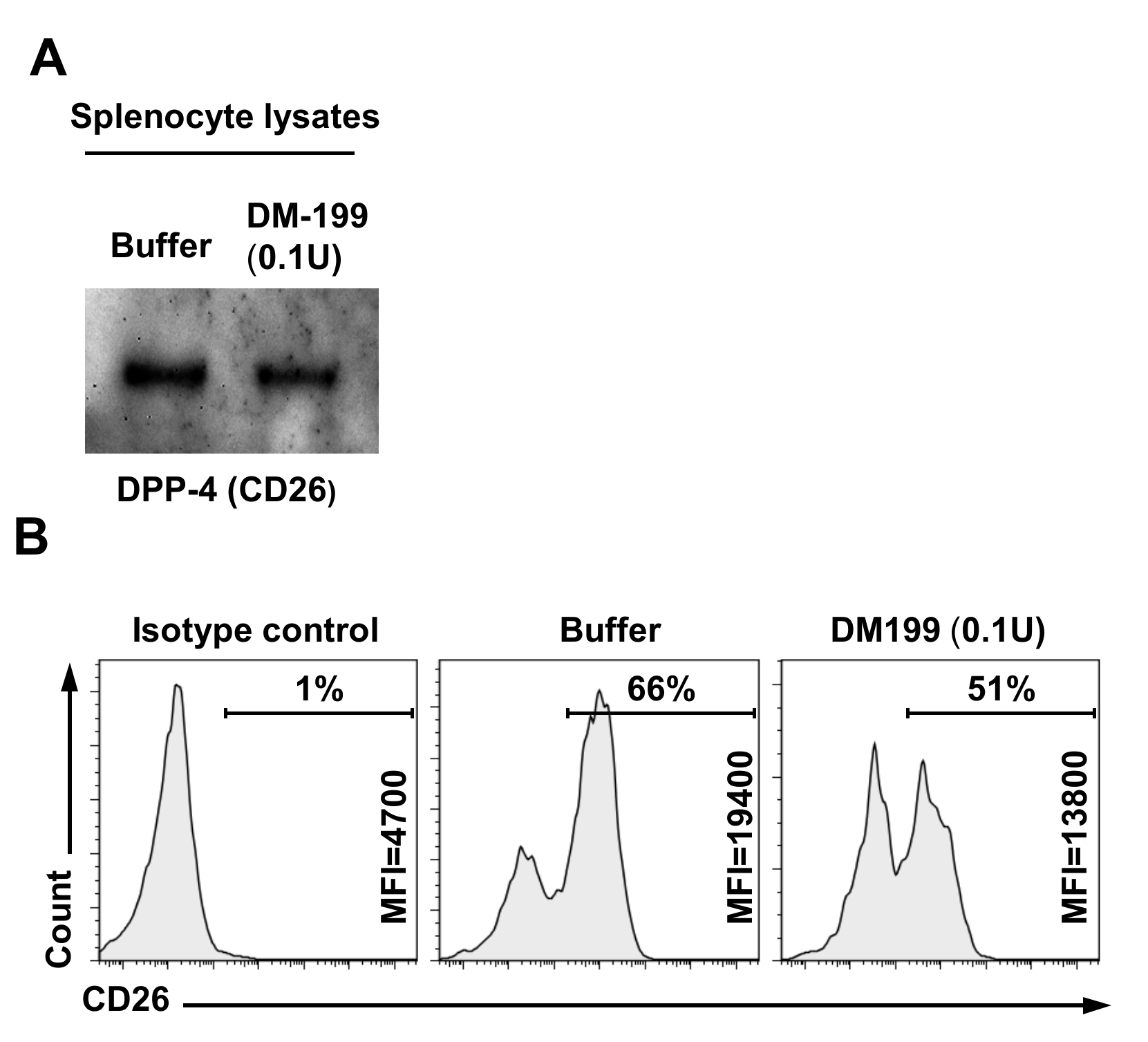

Supplement: Figure S2 — DM199 reduces cellular CD26 levels. Isolated splenocytes from mice were incubated for 2 h at 37°C in the absence (Buffer) or presence of 0.1 Units of DM199. (A) Western blot analysis. Splenocyte lysates were subject to electrophoresis, transferred to nitrocellulose membranes and probed with anti-DPP-4/CD26 rat monoclonal antibody. (B) FACS analysis. 1×10^5^ splenocytes treated with buffer or DM199 were stained with either isotype control antibody or FITC-labeled rat anti-CD26 and 7AAD prior to FACS analysis. MFI, mean fluorescence intensity. (TIF) [file pone.0107213.s002.tif]

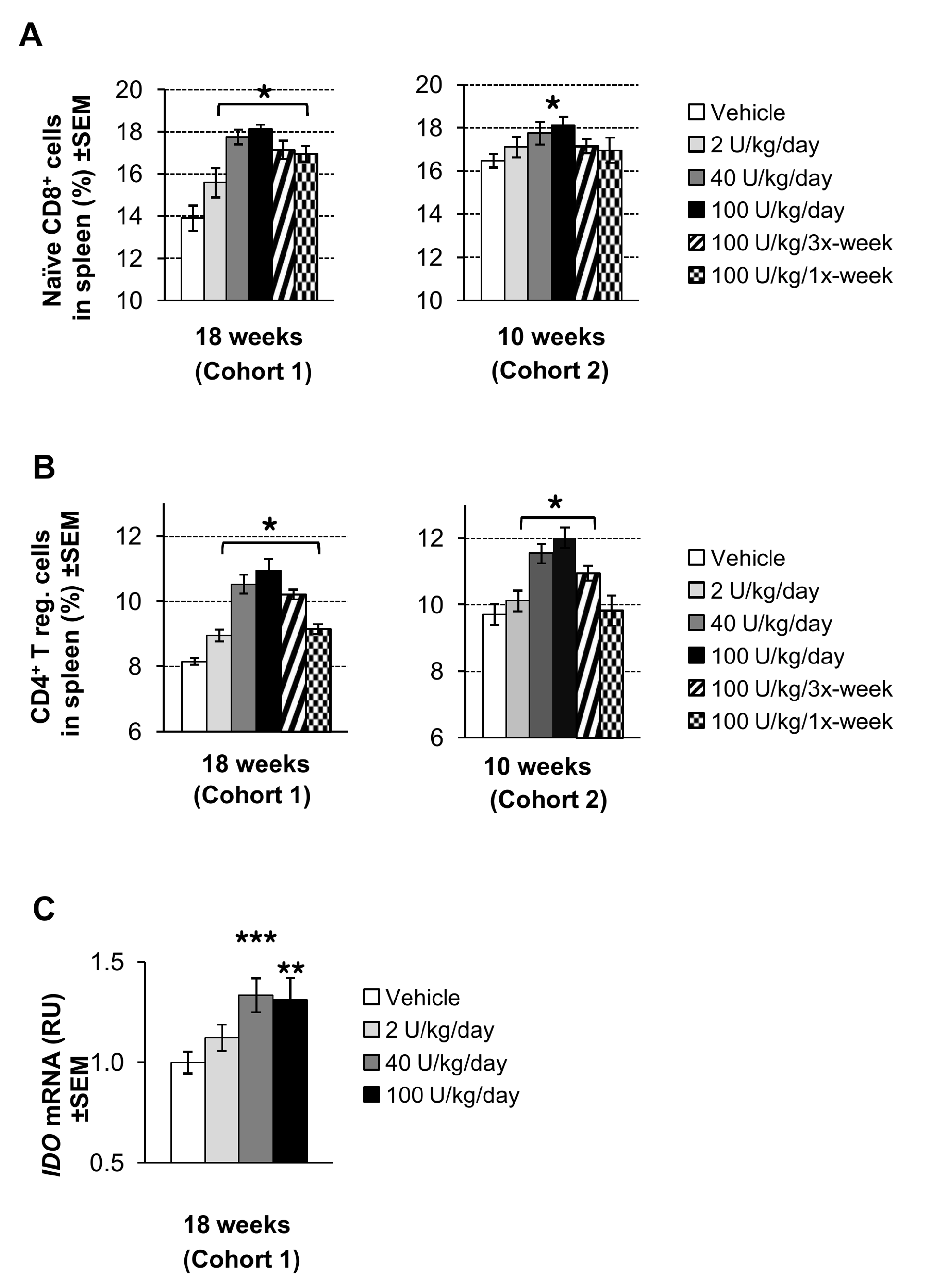

Supplement: Figure S3 — Immunomodulatory effects of DM199 treatment in the spleen. Single cell suspensions of splenic cells were incubated with ACK lysis buffer to remove erythrocytes, resuspended, stained with antibodies, and analyzed by FACS. (A) The percentage of CD44−CD8+ cells (naïve CD8+ cells) within the CD3+ T cell population is represented as mean ± SEM (n = 4–10 mice/group). (B) The percentage of CD4+CD25+Foxp3+ cells (T regulatory cells) within the CD3+ T cell population is represented as mean ± SEM (n = 4–10 mice/group). (C) Indoleamine 2,3-deoxygenase (IDO) mRNA expression in splenic dendritic cells after 10 weeks of treatment was determined by qPCR. Data in arbitrary units (AU) normalized to β-actin are represented as mean ± SEM (n = 5 mice/group). **P<0.05; ***P<0.001 vs. control using a one-way ANOVA with Tukey post-hoc multiple comparisons test. (TIF) [file pone.0107213.s003.tif]
